# Comparative Pharmacokinetics of Naringin in Rat after Oral Administration of Chaihu-Shu-Gan-San Aqueous Extract and Naringin Alone

**DOI:** 10.3390/metabo3040867

**Published:** 2013-09-30

**Authors:** Shu-Qi Li, Shu Dong, Zhi-Heng Su, Hong-Wu Zhang, Jing-Bo Peng, Chang-Yuan Yu, Zhong-Mei Zou

**Affiliations:** 1 Institute of Medicinal Plant Development, Chinese Academy of Medical Sciences and Peking Union Medical College, Beijing 100193, China; E-Mails: lishuqibrezz@163.com (S.-Q. L.); book_000@163.com (S.D.); hwzhang@implad.ac.cn (H.W.Z.); tinghai10142000@yahoo.com.cn (J.-B.P.); 2 College of Life Science and Technology, Beijing University of Chemical Technology, Beijing 100029, China; 3 Tianjin Tianshi College, Tianjin 301700, China; 4 Internal Trade Food Test & Scientific Research Institute, Beijing 100070, China; 5 Guangxi Medical University, Nanning 530021, China; E-Mail: suzhiheng1981@126.com

**Keywords:** pharmacokinetics, naringin, Chaihu-Shu-Gan-San, LC-MS/MS

## Abstract

Chaihu-Shu-Gan-San (CSGS), a traditional Chinese medicine (TCM) formula containing seven herbal medicines, has been used in the clinical treatment of gastritis, peptic ulcer, irritable bowel syndrome and depression in China. In order to explore the interaction between naringin and other constituents in CSGS, the pharmacokinetic difference of naringin in rats after oral administration of CSGS aqueous extract and naringin alone was investigated. The pharmacokinetic parameters of naringin in rats were achieved by quantification of its aglycone, naringenin by LC-MS/MS method. The double peaks phenomenon was observed in both serum profiles of rats after orally administered CSGS aqueous extract and naringin alone. However, the T_1/2__β_ was significantly decreased in rats given CSGS aqueous extract compared with naringin alone, and the mean residence time (MRT) and the area under the serum concentration–time curve (AUC_0-τ_) were higher than those of naringin, which indicated that naringin in CSGS had higher bioavailability, longer term efficacy and somewhat faster metabolism and excretion than those of naringin. The results suggested that certain ingredients co-exist in CSGS could influence pharmacokinetic behavior of naringin. This also provides a reference for human studies.

## 1. Introduction

Traditional Chinese Medicines (TCMs) have been proved to have a significant effect in the treatment of chronic and systematic diseases with fewer side effects than other treatments. In Chinese herbal therapy, the most widely used medicines are combined by many herbs and prepared according to TCM formulation concepts. It is acknowledged that complex interactions could produce synergistic effects and reduce possible side effects from some of the herbs. However, little evidence is available to interpret the mechanisms of those magic actions. Pharmacokinetics is useful to explain the metabolism status of drugs *in vivo* and to predict situations related to pharmacodynamics. Thus, pharmacokinetic characteristics of herbal medicines maybe benefit the interpretation of the rationality for the advantage of multi-constituents.

Chaihu-Shu-Gan-San (CSGS) is one of the most widely used TCM formulas in China for treatment of gastritis, peptic ulcer, irritable bowel syndrome and depression [1]. Pharmacological studies have proved that CSGS had prominent effects in various kinds of anti-inflammatory, antidepression, anti-ulcer, prevention of liver injury, and antioxidant [2]. CSGS is composed of seven commonly used Chinese herbs, *i.e.*, the roots of *Bupleurum chinense* DC. (Chai-Hu), the pericarps of *Citrus reticulata* Blanco (Chen-Pi), the roots of *Paeonia lactiflora* Pall. (Bai-Shao), the fruits of *Citrus aurantium* L. (Zhi-Qiao), the roots of *Cyperus rotundus* L. (Xiang-Fu), the roots of *Ligusticum chuanxiong* Hort. (Chuan-Xiong) and the roots of *Glycyrrhiza uralensis* Fisch. (Gan-Cao). Recently, the chemical profile of CSGS was investigated [3], 33 chemical constituents in CSGS were identified by LC-MS/MS and nine compounds including naringin, hesperidin, and neohesperidin were considered as major contributors to the antioxidant activity of CSGS.

The metabonomics study suggested that the antidepressant effect of CSGS could involve in regulating the dysfunctions of multiple metabolic pathways [4]. Recently, a new strategy using a combination of metabonomics and chemical profile was proposed to discover the chemical constituents which contribute to the antidepressant effect of CSGS [5]. As a result, naringin was demonstrated as one of the active constituents. However, up to now, no study on the pharmacokinetic characteristics of any constituents after oral administration of CSGS has been reported. In order to explore interactions among the multi-constituents in CSGS and its pharmacokinetic features, the pharmacokinetic difference of naringin in rats after oral administration of CSGS aqueous extract and naringin alone was investigated.

Oral absorption of naringin from citrus products suggested that naringin was poorly absorbed from the gastrointestinal tract in its original form [6]. The free form of naringin was transiently present in the plasma in rat and human and the glucuronide of naringenin were the predominant metabolite [7,8]. This paper describes a highly sensitive LC–MS/MS assay for the determination of naringenin glucuronides in rat serum. The validated method was applied to investigate the pharmacokinetic difference of naringin in rats after orally administrated CSGS aqueous extract and naringin alone, which will be helpful for understanding the interactions between multi-constituents in CSGS and the underlying mechanism of the synergistic effects.

## 2. Results and Discussion

### 2.1. Experimental Conditions Optimization

After the rats were orally administrated of CSGS aqueous extract or naringin monomer, the main present form in serum was naringenin glucuronide conjugation. So the concentration of naringin in rat serum can be expressed by the concentration of naringenin glucuronide conjugation, which was determined by a liquid chromatography system coupled to a mass spectrometry detector (MS/MS) after hydrolysis with *β*-glucuronidase. However, full-scan LC-MS/MS mass spectra cannot obtain obvious signals of the target component due to its quite low level content in biological samples. The multiple-reaction-monitoring (MRM) mode was preferable to distinguish target compound in complicated sample using precursor/product ion pairs under optimized mass conditions. The components can be identified even if they were not totally separated under the MRM detected mode.

Quercetin had resembled product ions and close retention times as naringenin in ESI-MS/MS, but they could be differentiated from each other in the MRM mode. Meanwhile, their ion pairs could reach highest abundance and similar sensitivity under the optimized MS conditions. Thus, quercetin qualifies as an internal standard (IS) for quantitative analysis of naringenin.

**Figure 1:**
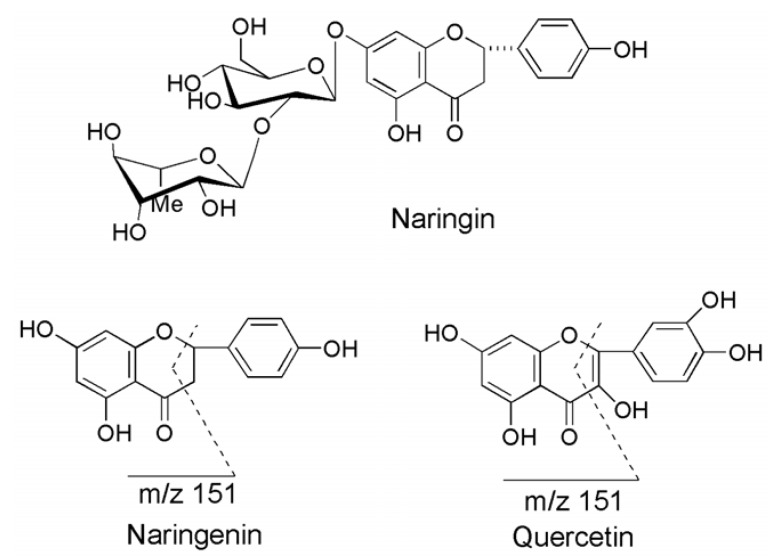
The chemical structures of naringin (NA), narigenin (NG) and quercetin (QU, IS).

In negative ion mode the precursor/product ion pairs of naringenin and IS were selected at *m/z* 271.1/151.1 and 301.2/151.2 (their possible product ion fragments were shown in Figure 1.) via syringe pump injection. Simultaneously, corresponding parameters DP, EP, CE, CXP and CEP were optimized to get highest abundance, sensitivity and stability. Then, the ion source was connected with HPLC system using the MRM method that had already been established to go on optimizing other parameters CUR Gas, CAD Gas, IS, TEM, GS1 and GS2. Thus, all the parameters were determined as the data were described in LC/MS/MS Apparatus and Chromatographic Conditions.

### 2.2. Method Validation

The assay method was validated for selectivity, stability, carry-over, linearity, precision, and accuracy.

**Figure 2:**
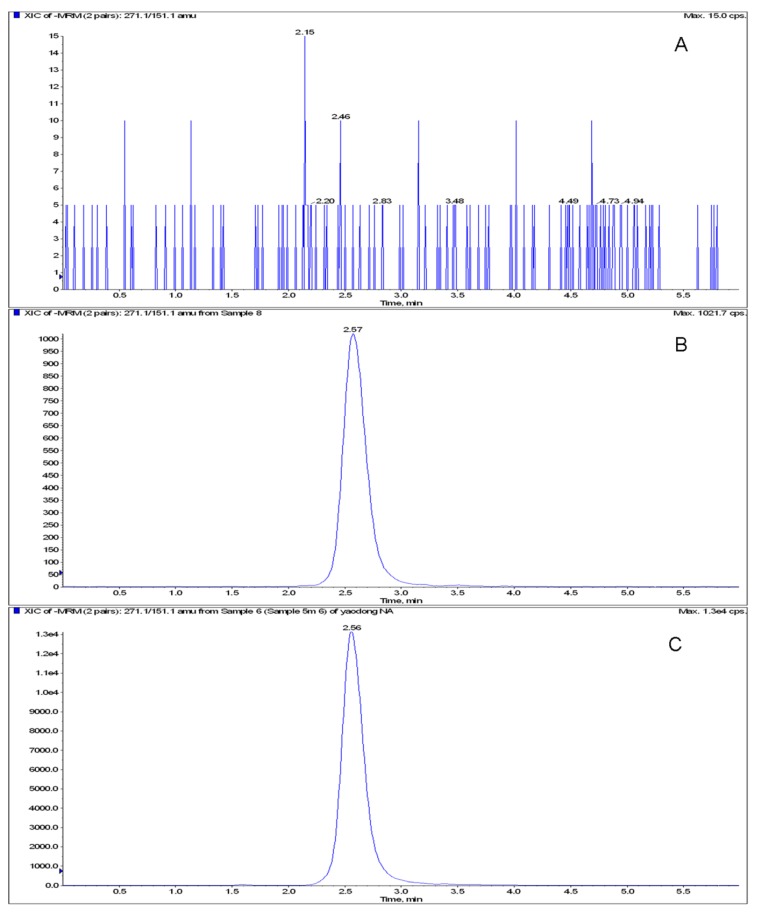
The multiple-reaction-monitoring (MRM) chromatograms of rat serum monitored at *m/z* 271.1/151.1. (**A**) Blank serum. (**B**) Blank serum spiked with naringenin. (**C**) Serum sample after administration of naringin.

**Figure 3:**
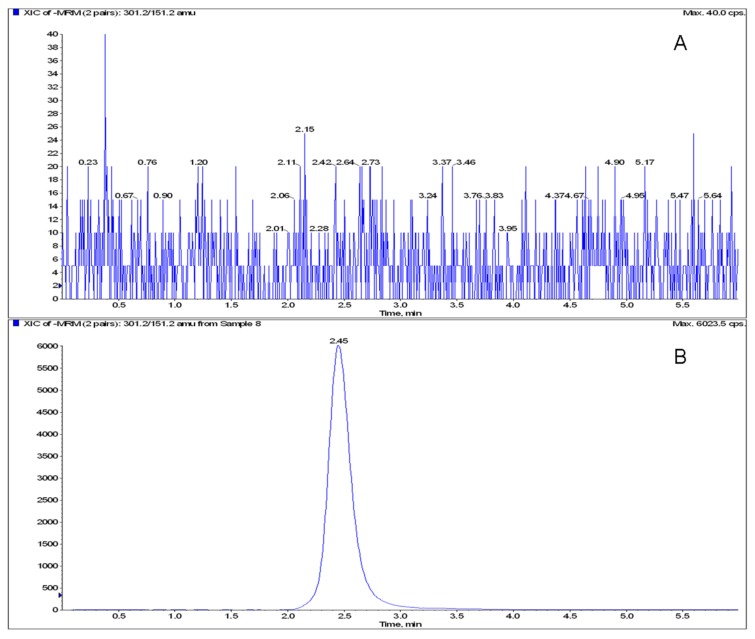
The MRM chromatograms of blank serum monitored at *m/z* 301.2/151.2. (**A**) Blank serum. (**B)** Blank serum spiked with quercetin.

The MRM chromatograms of blank serum, blank serum spiked with naringenin and quercetin, serum sample of rat after administrating naringin were shown in Figure 2 and Figure 3. There was no significant endogenous interference at the region of naringenin and quercetin in blank rat serum.

The extraction recoveries from QC samples at low, middle and high concentrations were (77.84 ± 3.73)%, (83.94 ± 3.50)% and 90.16 ± 4.66% for naringenin, whereas 86.08% ± 4.86 for IS. The data indicated that liquid-liquid extraction method in sample preparation was quite acceptable.

The calibration curve was linear over the concentration range of 0.036-18 *μ*g·mL^-1^ of naringenin Mean linear regression equation was *Y* = (7.786 ± 0.324)*X* − (0.0778 ± 0.0452) with regression coefficient r ≥ 0.9993. LOQ (S/N ≥ 10) of the method was 0.036 *μ*g·mL^-1^ and LOD (S/N ≥ 5) was 0.0225 *μ*g·mL^-1^.

Precision is represented as the relative standard deviation (%R.S.D.) and accuracy was calculated as the relative error (%R.E.) from the respective nominal concentration. The maximum acceptable limit for precision and accuracy was set at 15%. The data of intra- and inter-day precision and accuracy for naringin and naringenin from QC samples are summarized in Table 1. The results indicate an acceptable precision and accuracy of the present method.

**Table 1:** Intra-day and Inter-day precision of narigenin in rat plasma (n = 5).

Spiked concentration (*μ*g·mL^-1^)	Intra-day			Inter-day		
	Measured concentration (*μ*g·mL^−1^)	Precision (%)	Accuracy (%)	Measured concentration (*μ*g·mL^−1^)	Precision (%)	Accuracy (%)
0.36	0.392	3.27	104.00	0.423	7.78	105.17
	0.365			0.347		
	0.372			0.389		
	0.381			0.375		
	0.362			0.359		
	0.374 ± 0.012			0.379 ± 0.02		
1.8	1.82	3.77	99.00	1.90	5.3	98.56
	1.77			1.83		
	1.86			1.79		
	1.78			1.64		
	1.68			1.71		
	1.78 ± 0.07			1.77 ± 0.10		
9.0	8.96	1.22	100.69	9.08	2.18	99.89
	9.06			8.72		
	9.24			9.24		
	8.98			9.02		
	9.07			8.89		
	9.06 ± 0.11			8.99 ± 0.20		

The stability data are summarized in Table 2 and indicated that the serum samples at three concentrations kept at −80 °C for 15 days and QC samples at three concentrations exposed to 10 hours at temperature conditions were all stable.

**Table 2:** Stability of quality control (QC) samples.

Concentration (μg·mL^−1^)	Short term (at room temperature)					Long term (at −80 °C)					Freeze-thaw (cycle number)				
	0	4h	6h	10h	RSD (%)	0	5d	10d	15d	RSD (%)	0	1	2	3	RSD (%)
0.36	0.342	0.354	0.374	0.365	3.85	0.347	0.365	0.418	0.384	8.02	0.357	0.403	0.382	0.369	5.17
1.8	1.87	1.86	1.78	1.80	2.30	2.02	1.95	1.79	1.87	5.21	1.78	1.89	1.92	1.82	3.45
9	9.94	9.87	9.29	10.03	3.42	9.07	9.47	9.24	9.05	2.11	8.90	9.23	9.89	9.09	4.64

The extraction recovery data are summarized in Table 3 and indicated that the serum samples at three concentrations using five replicates are fully extracted.

**Table 3:** Extraction recoveries of NG and QU control samples (n = 5).

	Concentration ( *μ*g·mL^−1^)	Extracted peak area	Control peak area	Absolute recovery (%)	Average recovery (%)	RSD (%)
NG QC	0.36	2.08e + 003	3.17e + 003	78.74	77.84	4.80
		2.13e + 003	3.22e + 003	79.38		
		2.18e + 003	3.64e + 003	71.87		
		2.28e + 003	3.34e + 003	81.92		
		2.08e + 003	3.23e + 003	77.28		
	1.8	2.15e + 004	3.12e + 004	82.69	83.94	4.17
		2.07e + 004	3.05e + 004	81.44		
		2.22e + 004	3.08e + 004	86.49		
		1.99e + 004	2.97e + 004	80.40		
		2.29e + 004	3.10e + 004	88.64		
	9	1.97e + 005	2.58e + 005	91.63	90.16	5.17
		1.67e + 005	2.42e + 005	82.81		
		1.65e + 005	2.23e + 005	88.79		
		1.93e + 005	2.44e + 005	94.92		
		1.83e + 005	2.37e + 005	92.66		
QU (IS)	5	6.34e + 004	8.23e + 004	92.44	86.08	5.65
		5.98e + 004	9.02e + 004	79.56		
		5.81e + 004	8.32e + 004	83.80		
		5.87e + 004	8.19e + 004	86.01		
		6.38e + 004	8.64e + 004	88.61		

### 2.3. Pharmacokinetic Study

**Figure 4:**
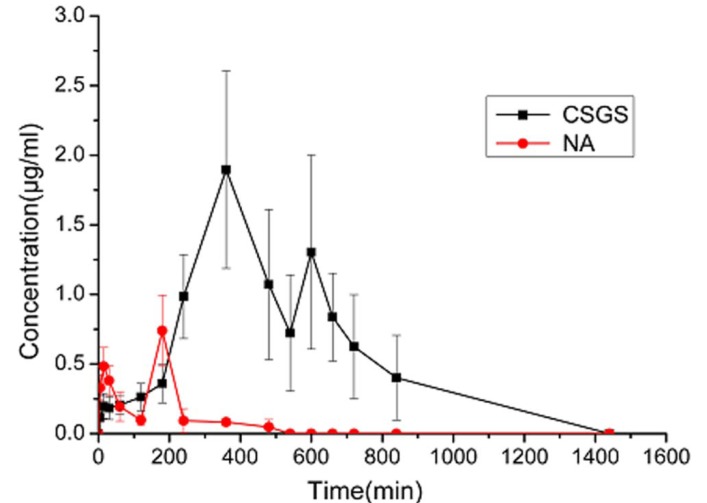
Serum concentration-time curve of naringenin in rats after orally administration of Chaihu-Shu-Gan-San and naringin.

The pharmacokinetic study of naringin in rats via oral administration of CSGS aqueous extract and naringin monomer were carried out by using the validated method. The serum concentration-time profiles were shown in Figure 4. Double peak phenomenon was both appeared in intake of naringin alone and CSGS aqueous extract, but the times of the peaks were quite different. Pharmacokinetics was analyzed using the non-compartmental model of WinNonlin and the main pharmacokinetic parameters were summarized in Table 4.

**Table 4:** Non-compartmental model pharmacokinetic parameters of rats after orally administered Chaihu-Shu-Gan-San (CSGS) aqueous extract and naringin monomer.

Parameters	Units	CSGS	NA
Tmax,1	min	360.0000	15.0000
Cmax,1	mg·L^-1^	1.8970	0.4840
Tmax,2	min	600.0000	180.0000
Cmax,2	mg·L^-1^	1.3040	0.7390
Lambda_z	1·min^-1^	0.0040	0.0027
HL_Lambda_z	min	171.8932	255.7001
AUCall	min·mg·L^-1^	840.2175	114.0243
Cl_F	L·min^-1^·kg^-1^	3.0512	0.0443
MRT	min	523.3516	274.8070

As shown in Figure 4, naringin was quickly absorbed into serum by reaching the first concentration peak at 15 min and another at 3 h after orally administered naringin monomer. After dosing for 480 min, it could not be detected, which may due to the fast metabolism (5.075 mg·kg^-1^). The AUC_all_ and MRT were 274.8070 min×mg·L^-1^ and 114.0243 min. Pharmacokinetic study [8] of naringenin in rats showed that the total naringenin content existed dose-dependent phenomenon. AUC linearly increased as the dose rose. The higher dosage the rats received, the longer time it took to reach T_max_ and the longer of the MRT.

Compared with naringin monomer, the T_max,1_ was at 6h after administration of CSGS. The AUC_all_ and MRT were 840.2175 min×mg·L^-1^ and 523.3516 min, which indicated that naringin in CSGS had high bioavailability and long term efficacy. There was a shorter terminal elimination half-life (HL_Lambda_z) at 171.8932 min, which may explain why, after absorption, the latter metabolism of distribution and excretion were somewhat faster. It was also an indirect reflection that the metabolism state of naringin in CSGS was better than the naringin monomer with short time accumulation, fast distribution and small toxic and side-effects. Absorption was much slower, which confirmed that traditional Chinese medicine had a slower efficiency.

In accordance with other flavonoids [9,10], the present pharmacokinetics of naringin monomer took on double peaks. Moreover, the main circulated form of these flavonoids was the conjugated metabolites [10,11]. Conjugation increased solubility of compounds and played an important role in excretion [12]. Flavonoid glucuronides are likely to be excreted in the bile because of the increased molecular weight and polarity. Then they can be hydrolyzed to the free form for reabsorption by the bacterial *β*-glucuronidases via enterohepatic circulation, which may be one cause of double peaks. Study of naringenin metabolism in rats [8] confirmed that its glucuronides excreted in bile then underwent enterohepatic circulation after absorption and pharmacokinetics of naringenin showed double peak phenomenon. In the present study, metabolism of naringin and the phenomenon of two peaks may be explained as follows: the rats were orally administered naringin, which was hydrolyzed by intestinal bacteria to naringenin or partly hydrolyzed to naringenin glucuronides [13,14], then, the metabolites were absorbed and some of naringenin could recombine with free glucuronides. The conjugated derivatives were absorbed into blood and circulated in the body to distribute into tissues, while in bile the conjugates excreted. After enterohepatic circulation, naringenin was reabsorbed into blood.

Double peak phenomenon of drug concentration-time curves may result from many factors related to metabolism *in vivo* and is a complicated phenomenon to be explained, especially for traditional Chinese medicine recipes [15]. In CSGS, metabolism of naringin was quite different compared with naringin monomer, which may because of the complexity and multiple components of the recipe. CSGS was rich in flavonoids (equal to 42.28 ± 1.80 mg rutin per gram aqueous extract of CSGS in our previous experiment). Competitive inhibition at the stage of reabsorption between naringin and these ingredients that might undergo a similar metabolic way could decrease the hydrolysis reaction of naringenin conjugates due to a limited amount of intestinal bacteria and enzymes. Therefore, the amount of naringin which underwent enterohepatic circulation was decreased and its terminal elimination half-life was shortened. That may be why the HL_Lambda_z of CSGS was shorter than the naringin monomer and the concentration at the second peak was lower than the first one, while the naringin monomer showed the opposite results. Enterohepatic circulation was considered to be associated with side-effects [16]. After rats were orally administered anti-inflammatory drug flufenamic acid, the elimination half-life in plasma was 7.5 h with high rate of ulceration occurrence. However, in the bile duct ligated rats, the elimination half-life was shortened to 5.1 h with no ulceration occurring [16]. Naringin in CSGS had shorter terminal elimination half-life, showing the advantage of the Chinese medicinal recipe with low toxic side-effects.

Besides complicated chemical circumstances in the organism, there are other factors that may affect the metabolic state of the medicinal recipe. The various chemical components in the prescription itself and the combination of herbs such as herb-herb interaction and component-herb interaction may affect the metabolism. The exact mechanism is still unclear and needs to be studied further. In our study we found that naringin in CSGS had a slow action after dosing. However, the bioavailability was enhanced, the efficacy term was prolonged, the elimination half-time was shortened, and the side-effect was reduced, which confirmed the nationality of herbs in CSGS combined in ratio and used as a whole recipe.

According to the data [17], after intravenous and oral administration, the pharmacokinetic parameters of two enantiomers of naringenin did not show significant differences except that AUCiv of R-naringenin was 7.002 μh·mL^-1^ (35%) higher than that of S form. Therefore, naringin metabolized into naringenin, an enantiomer of naringenin, did not affect their pharmacokinetic parameters.

## 3. Experimental Section

### 3.1. Chemicals and Reagents

Naringin, naringenin and quercetin were obtained from National Institute for the Control of Pharmaceutical and Biological Products (Beijing, China). *β*-glucuronidase (Ⅸ-A from *E.Coli*) was purchased from Sigma-Aldrich (Shanghai, China). Acetonitrile and formic acid (Fisher Scientific, Waltham, MA, USA) were of HPLC grade. Water was produced by Milli-Q water system (Millipore, Christiansburg, VA, USA). All other chemicals were analytical grade and obtained from Beijing Beihua Fine Chemical Co., Ltd. (Beijing, China).

### 3.2. Preparation of CSGS Aqueous Extract

CSGS contains seven different herbal medicines that are the root of *Bupleurum chinense* DC. (Chai-Hu), Paeonia lactiflora Pall. (Shao-Yao), Cyperus rotundus L. (Xiang-Fu), Ligusticum chuanxiong Hort. (*Chuan-Xiong*), Glycyrrhiza Uralensis Fisch. (Gan-Cao), the pericarp of Citrus reticulata Blanco. (Chen-Pi) and Citrus aurantium L. (Zhi-Qiao). All raw herbs were provided by Tong-Ren-Tang Traditional Herbal Medicine Company (Bejing, China). Their identities were verified by Associate Prof. Yu-Lin Lin of Institute of Medicinal Plant Development, Chinese Academy of Medical Sciences, China. The voucher specimens are deposited in the Natural Medicine Research Center of this Institute.

The CSGS aqueous extract was prepared as previously described [2]. Five hundred grams of mixed crude herbs, Chai-Hu, Chen-Pi, Shao-Yao, Zhi-Qiao, Xiang-Fu, Chuan-Xiong and *Gan-Cao* were mixed in the proportion of 4:4:3:3:3:3:1 and were soaked together in water (*w*/*v*: 1 g: 10 mL) for 30 min at room temperature and then decocted for 2 h. After collecting the filtrate, the herbs were then decocted in the same volume of water for an additional 2 h. The filtrates were combined and concentrated in vacuum to obtain 39 g of CSGS aqueous extract (yield: 7.8%). One gram of CSGS aqueous extract contains 2.03 mg of naringin.

### 3.3. Animals

Male Wistar rats (body weight 200 ± 25 g) were purchased from Vital River Laboratory Animal Technology Co. Ltd. (Beijing, China). The animals were kept under controlled conditions (temperature: 23 ± 2 °C; humidity: 55 ± 5%) for one week acclimation before experiment and were allowed free access to standard laboratory diet and water during the period. The breeding and experimental protocol was in accordance with the ethical principles of animal use and care outlined by Ethics Committee of the Institute of Medicinal Plant Development, CAMS&PUMC.

### 3.4. LC/MS/MS Apparatus and Chromatographic Conditions

HPLC analysis was carried out using an Agilent Technolofies1200 Series HPLC system (Agilent: Santa Clara, California, CA, USA), including quaternary pump (Agilent 1200 G1311A), diode array detector (Agilent 1200 DAD G1315B), autosampler (Agilent 1200 G1329A) and thermostatted column oven. Separation was performed on a Venusil ASB-C18 column (5 μm, 2.1 × 150 mm; Agela Technologies, Wilmington, DE, USA) at room temperature. The mobile phase was acetonitrile—1% formic acid (30:70, v/v) at a flow rate of 0.2 mL·min^-1^. 10 μL of the sample was injected for each analysis with a period of 6 min run time.

MS analysis was performed with API 3200 Triple Quadrupole mass spectrometer (Applied Biosystems: Minneapolis, Minnesota, USA) equipped with Turbo Ionspray Source. Quercetin was used as the internal standard (IS). The system was operated in electrospray negative ionization using MRM (multiple reaction monitoring) mode monitoring the transition of *m/z* 271.1→151.1 and *m/z* 301.2→151.2 for naringenin and quercetin (IS), respectively. The optimized parameters are as follows: Declustering potential (DP, V): –65; Entrance potential (EP, V): –10; Collision energy (CE, V): –30; Collision cell entrance potential (CEP, V): –10; Collision cell exit potential (CXP, V): –16; Curtain gas (nitrogen) (CUR Gas, psi): 45; Collision gas (CAD Gas): Medium; Ion spray voltage (IS, V): –4500; Source temperature (TEM, °C) 600; Ion source gas1 (GS1, psi): 50; Ion source gas 2(GS2, L·min^-1^): 10; Dwell time per transition (ms) 200. Data was collected and processed using Analyst software 1.4.2 (AB/MDS/SCIEX).

### 3.5. Preparation of Calibration and Quality Control (QC) Samples

The standard stock solution of 90 μg·mL^-1 ^ of naringenin was prepared in acetonitrile-water (10:90 V/V). A series of standard solutions with concentration in the range of 0.045–22.5 mg·mL^-1^ were obtained by further dilution of the standard stock solution with acetonitrile-water (10:90 *V*/*V*). The internal standard stock solution of 1 mg·mL^-1^ of quercetin was prepared in acetonitrile. Internal standard working solution (20 μg·mL^-1^) was prepared by diluting the internal standard stock solution with acetonitrile. All solutions were stored at 4 °C. To prepare the calibration samples, 80 *μ*L of working solutions and 30 μL of IS were diluted with 100 μL blank serum to span a calibration standard range of 0.036–18 μg·mL^-1^ (0.036, 0.36, 1.8 4.5, 9, 18 μg·mL^-1^). Quality control (QC) samples (0.36, 1.8, 9 μg·mL^-1^) were prepared in the same way and were stored at 4 °C until analysis.

### 3.6. Preparation of Serum Samples

An aliquot of 100 μL of serum sample was incubated with 30 μL of β-glucuronidase solution (dissolved in phosphatic buffer pH = 6.8, 199.2 units·mL^-1^) at 37 °C for 2 h. 50 μL of 0.1 N HCl and 30 μL of IS solution (20 μg·mL^-1^) were added in turn, then vortexed thoroughly. The samples were extracted with 600 μL of ethyl acetate by vortex-mixing for 1 min. After centrifugation at 10000 rpm for 3 mins (4 °C), 500 μL of the upper (organic) layer was transferred to a test tube and evaporated to dryness under a stream of nitrogen. The residue was reconstituted in 100 μL of mobile phase and stored at 4 °C until use.

### 3.7. Method Validation

The method was validated for selectivity, linearity, precision, accuracy, extract recovery and stability. Validation runs were conducted on three consecutive days. The peak area ratio of naringenin to the IS of QC samples were interpolated from the calibration curve on the same day to give concentrations of the two analytes. The results from QC samples in three runs were used to evaluate the precision and accuracy of the method developed.

#### 3.7.1. Specificity

Specificity was assessed by analysis of six different samples of blank matrix with and without spiking with naringenin and IS.

#### 3.7.2. Calibration Curve and Sensitivity

Calibration curves were constructed from working standard solutions of naringenin at concentration range 0.036–18 μg·mL^-1^ by plotting peak-area ratio of naringenin to the internal standard, versus naringenin concentration Linearity was assessed by weighted linear regression of calibration curves generated in triplicate on three consecutive days using analyte-internal standard peak area ratios. Unknown sample concentrations of naringenin in serum samples were calculated from the linear regression equation for the calibration plot of peak area ratio against concentration. The limit of detection (LOD) was defined as a signal to noise ratio of 5:1. The lower limit of quantification (LLOQ) was determined in accordance to the base line noise, considering a signal-to-noise ratio of 10:1.

#### 3.7.3. Accuracy and Precision

The accuracy and precision of the established method were evaluated by QC samples at low, medium and high concentrations as mentioned above. The concentration of each QC sample was calculated using calibration curves prepared each day. Accuracy was defined as the relative deviation in the calculated value (E) of a standard from that of its true value (T), expressed as relative error (R.E.). The intra-day accuracy was determined by assaying five replicates at each concentration level on 1 day, and inter-day accuracy was determined by analyzing QC samples in duplicates during five successive days. Precision was evaluated as the relative standard deviation (R.S.D.).

#### 3.7.4. Extraction Recovery

The recovery of analyte and IS was determined by comparing the responses of the analyte from QC samples with the responses of analyte spiked in post-extracted blank rat serum at equivalent concentrations using five replicates. The recovery of IS was determined at a single concentration of 5 μg·mL^-1^.

#### 3.7.5. Stability

Stability of naringenin in serum was evaluated after sample extraction process (QC samples were processed and stored under autosampler condition for 10 h), three freeze-thaw cycles and long-term freezing at −80 °C (14 days) by QC samples in six replicates at each concentration. Stability was assessed by comparing the mean concentration of the stored QC samples with the mean concentration of freshly prepared QC samples. Stability samples were to be concluded stable if the bias of them was within ±15% of the actual value.

### 3.8. Pharmacokinetic Study

All the rats were fasted overnight with free access to water and randomly divided into two groups, and each group was single dose administered CSGS aqueous extract (2.5 g·kg^-1^ B.W.) or NA (5.075 mg·kg^-1^ B.W.) via oral gavage. Administered dosage of naringin was calculated from the content contained in CSGS (2.03 mg naringin /g CSGS). CSGS aqueous extract and naringin powder were dissolved in 50% PEG 400. Approximately 1 mL blood was withdrawn from eye vein at 0, 5, 15, 30, 60, 120, 180, 240, 360, 480, 540, 600, 660, 720, 840 and 1,440 min after dosing. Blood samples were centrifuged at 10000 rpm for 10 min at 4°C to obtain the serum which was then stored at −80°C until analysis.

Pharmacokinetic analysis was carried out using WinNonlin software (Version 5.2.1, Pharsight Corporation, Mountain View, CA, USA) with a noncompartmental model.

## 4. Conclusions

In the study, a specific LC-MS/MS method was built up and was validated and appropriate for quantitative analysis of naringenin in rat serum. Pharmacokinetics of naringin alone and in CSGS were studied and the differences in serum concentration profiles and pharmacokinetic parameters indicated that metabolism of monomer ingredient was greatly affected in prescriptions. The results also showed that Chinese medicinal recipes had many advantages such as long term efficacy, high bioavailability and low toxic side-effects, which the monomer could not measure up to. Furthermore, our study provided valuable information for understanding the interactions of multi-constituents and synthetic effects of herbal combination in prescriptions.
